# Intestine-Specific Deletion of Microsomal Triglyceride Transfer Protein Increases Mortality in Aged Mice

**DOI:** 10.1371/journal.pone.0101828

**Published:** 2014-07-10

**Authors:** Zhe Liang, Yan Xie, Jessica A. Dominguez, Elise R. Breed, Benyam P. Yoseph, Eileen M. Burd, Alton B. Farris, Nicholas O. Davidson, Craig M. Coopersmith

**Affiliations:** 1 Emory Center for Critical Care and Department of Surgery, Emory University School of Medicine, Atlanta, Georgia, United States of America; 2 Department of Medicine, Washington University School of Medicine, St. Louis, Missouri, United States of America; 3 Department of Basic Sciences, Bastyr University California, San Diego, California, United States of America; 4 Department of Pathology and Laboratory Medicine, Emory University School of Medicine, Atlanta, Georgia, United States of America; Charité, Campus Benjamin Franklin, Germany

## Abstract

**Background:**

Mice with conditional, intestine-specific deletion of microsomal triglyceride transfer protein (*Mttp-IKO*) exhibit a complete block in chylomicron assembly together with lipid malabsorption. Young (8–10 week) *Mttp-IKO* mice have improved survival when subjected to a murine model of *Pseudomonas aeruginosa*-induced sepsis. However, 80% of deaths in sepsis occur in patients over age 65. The purpose of this study was to determine whether age impacts outcome in *Mttp-IKO* mice subjected to sepsis.

**Methods:**

Aged (20–24 months) *Mttp-IKO* mice and WT mice underwent intratracheal injection with *P. aeruginosa*. Mice were either sacrificed 24 hours post-operatively for mechanistic studies or followed seven days for survival.

**Results:**

In contrast to young septic *Mttp-IKO* mice, aged septic *Mttp-IKO* mice had a significantly higher mortality than aged septic WT mice (80% vs. 39%, p = 0.005). Aged septic *Mttp-IKO* mice exhibited increased gut epithelial apoptosis, increased jejunal Bax/Bcl-2 and Bax/Bcl-X_L_ ratios yet simultaneously demonstrated increased crypt proliferation and villus length. Aged septic *Mttp-IKO* mice also manifested increased pulmonary myeloperoxidase levels, suggesting increased neutrophil infiltration, as well as decreased systemic TNFα compared to aged septic WT mice.

**Conclusions:**

Blocking intestinal chylomicron secretion alters mortality following sepsis in an age-dependent manner. Increases in gut apoptosis and pulmonary neutrophil infiltration, and decreased systemic TNFα represent potential mechanisms for why intestine-specific *Mttp* deletion is beneficial in young septic mice but harmful in aged mice as each of these parameters are altered differently in young and aged septic WT and *Mttp-IKO* mice.

## Introduction

Sepsis affects over 800,000 patients annually in the United States, and between 230,000 and 370,000 patients die of the disease annually [1]. Sepsis is a disease of the aged. Even though elderly patients account for slightly greater than 10% of the population in the United States, more than 60% of cases of sepsis and approximately 80% of deaths occur in patients over 65 years of age [2], [3]. Both animal and human studies demonstrate that age greatly impacts the host response following sepsis and not only is mortality higher with aging, but also multiple therapies that are effective in young mice are ineffective in aged mice [4]–[12]. Despite this, the vast majority of rodent models of sepsis use 6–16 week old animals, which corresponds to a 10–17 year old human [13]. It has been suggested that the use of young animals to model a disease of the aged – which have altered inflammation, coagulation, apoptosis, and proteomics – is one of many reasons why preclinical models of sepsis have failed to translate into positive clinical trials [14], [15].

The gut has long been hypothesized to be “the motor” of the systemic inflammatory response syndrome [16]–[19]. Critical illness worsens gut integrity in a number of ways, including increasing intestinal epithelial apoptosis and permeability while simultaneously decreasing both proliferation and mucous production [20]–[44]. The microbiome of the gut – which ordinarily exists in a symbiotic relationship with the host – can also respond to environmental cues in sepsis to induce virulence factors, which can, in turn, worsen critical illness [45]–[48]. Additionally, toxic gut-derived lymph induces distant organ injury in critical illness as numerous lines of evidence support the “gut lymph” hypothesis in which the intestine releases toxic mediators that are transported through the mesenteric lymph nodes and cause damage remote from the intestine [49]–[57].

The role of intestinal chylomicron assembly and secretion in transporting toxic gut-derived lipid factors is poorly understood. Microsomal triglyceride transfer protein (Mttp) resides within the lumen of the endoplasmic reticulum and plays a requisite role in the assembly of triglyceride-rich lipoprotein particles, both within enterocytes of the small intestine and also in hepatocytes of the liver [58]. We previously studied the impact of sepsis in young (8–10 week old) mice with defective chylomicron assembly induced by conditional intestine-specific deletion of Mttp (*Mttp-IKO* mice) [59]. Compared to young septic wild type (WT) mice, young septic *Mttp-IKO* mice have increased survival. This is associated with improvements in gut integrity, manifested by decreased gut apoptosis, increased crypt proliferation and longer villus length. Young septic *Mttp-IKO* mice also have marked decreases in systemic IL-6 and G-CSF. Despite pneumonia being initiated in the lungs, no significant pulmonary differences exist between young septic *Mttp-IKO* and WT mice, with similar levels of MPO activity, bacterial clearance and broncoalveolar lavage (BAL) cytokines. The aim of this study was to determine if aging alters the beneficial impact seen with intestine-specific *Mttp* deletion in young septic mice.

## Materials and Methods

### Animals

Aged (defined as 20–24 months old) Mttp^flox/flox^ villin-Cre-ER^T2^ (*Mttp-IKO*) mice on a background of ∼75% C57BL/6 and ∼25% 129/SvJ were utilized for all experiments. Conditional, intestine-specific deletion of Mttp was induced via Cre recombinase expression in villus epithelial cells in which animals received five daily injections of tamoxifen given intraperitoneally (1 mg, Sigma, St. Louis, MO) [59], [60]. Experiments were performed three weeks after tamoxifen injections. Age matched littermate animals that did not receive tamoxifen (i.e. functionally WT) were used as controls. All experiments were performed in accordance with the National Institutes of Health Guidelines for the Use of Laboratory Animals and were approved by the Institutional Animal Care and Use Committee at Emory University School of Medicine (Protocol DAR-2000539-121812). All surgery (described below) was performed under isoflurane anesthesia and all efforts were made to minimize animal suffering by treating all animals with buprenex post-operatively. For non-survival studies, animals were sacrificed 24 hours following intratracheal injection of P. *aeruginosa* (detailed below) using asphyxiation by CO2 or via exsanguination under deep isoflurane anesthesia. A different subset of animals was followed for 7 days after intratracheal injection of P. *aeruginosa* for survival studies. Animals were checked twice daily. Moribund animals were sacrificed using humane endpoints. The following criteria were used to identify moribund animals: a) major organ failure or medical conditions unresponsive to treatment such as severe respiratory distress, icterus, uremia, intractable diarrhea, or self-mutilation, b) surgical complications unresponsive to immediate intervention (bleeding, infection, wound dehiscence) or c) clinical or behavioral signs unresponsive to appropriate intervention persisting for 24 hours including significant inactivity, labored breathing, sunken eyes, hunched posture, piloerection/matted fur, one or more unresolving skin ulcers, and abnormal vocalization when handled. Note that it is important that many classical humane endpoints for animal studies are not sufficient for studies in sepsis, as many animals meeting general guidelines criteria for euthanasia survive despite sepsis. Specific guidelines for humane endpoints in sepsis research [61], [62] were followed. Non-moribund animals that died during the course of the survival curve died as a direct result of their septic insult. Animals that survived 7 days after intratracheal injection of P. *aeruginosa* were sacrificed at the conclusion of this experiment using asphyxiation by CO2.

### Sepsis model

Under isoflurane anesthesia, all animals were subjected to intratracheal injections of *P. aeruginosa* (ATCC 27853) via midline cervical incision [63], [64]. A total of 40 µl of bacteria diluted in normal saline (2–4×10^7^ CFU) was injected via a 29 gauge syringe. To enhance bacterial delivery, mice were held vertically for 10 seconds. All mice received a subcutaneous injection of 1 ml saline post-operatively to compensate for insensible fluid losses. Animals were either euthanized 24 hours post-operatively or followed 7 days for survival.

### Intestinal morphology

Villus length was measured as the distance in µm from the crypt neck to the villus tip. Crypt depth was measured as the distance in µm from the crypt base to crypt neck. Each was measured in 12–16 well-oriented jejunal villi and crypts using Nikon Elements imaging software- EIS-Elements BR 3.10 (Nikon Instruments, Melville, NY).

Intracellular lipid droplets were evaluated using osmium tetoxide staining in intestinal tissue fixed in 10% neutral buffered formalin. Sections were then transferred into 1% osmium tetroxide, rinsed in distilled water, incubated in 0.5% periodic acid, washed and then processed for paraffin embedding, followed by counterstaining with hematoxylin and eosin (H&E).

### Intestinal apoptosis

Apoptotic cells were quantified in the jejunum using two independent but complementary techniques: active caspase-3 staining and morphologic analysis of H&E-stained sections [27]. For caspase-3 staining, sections were deparaffinized, rehydrated, and incubated in 3% hydrogen peroxide for 10 minutes. Sections were then placed in Antigen Decloaker (Biocare Medical, Concord, CA) and heated in a pressure cooker for 45 minutes. After sections were blocked with 20% normal goat serum (Vector Laboratories, Burlingame, CA), they were incubated overnight with rabbit polyclonal anti-active caspase-3 (1∶100; Cell Signaling, Beverly, MA) at 4°C. The following day, sections were incubated with goat anti-rabbit biotinylated secondary antibody (1∶200; Accurate Chemical and Scientific, Westbury, NJ) for 1 hour followed by horseradish peroxidase (HRP)- labeled streptavidin (Accurate Chemical and Scientific) for 1 hour. Sections were then developed with diaminobenzidine and counterstained with hematoxylin.

Apoptotic cells were identified on H&E-stained sections via characteristic morphological changes including cell shrinkage with condensed and fragmented nuclei and then quantified in 100 well-oriented contiguous crypt-villus units.

### Intestinal proliferation

Mice were intraperitoneally injected with 5-Bromo-2′deoxyuridine (BrdU, 5 mg/ml diluted in normal saline; Sigma) 90 minutes prior to sacrifice to label cells in S-phase. Sections were deparaffinized, rehydrated, incubated in 1% hydrogen peroxide for 15 minutes, immersed in Antigen Decloaker (Biocare Medical) and heated in a pressure cooker for 45 minutes. Sections were then blocked with Protein Block (Dako, Carpinteria, CA) for 10 minutes, and incubated with rat monoclonal anti-BrdU overnight at 4°C (1∶500; Accurate Chemical & Scientific). The following day, sections were incubated with goat anti-rat biotinylated secondary antibody (1∶500; Accurate Chemical and Scientific) for 1 hour, followed by HRP labeled Streptavidin (Accurate Chemical and Scientific) for 1 hour and then developed with diaminobenzidine and counterstained with hematoxylin. BrdU-stained cells were quantified in 100 well-oriented contiguous crypts.

### Gene expression of apoptosis mediators

Total RNA was isolated from frozen jejunal tissue using the RNeasy Mini Kit (QIAGEN, Santa Clarita, CA) according to manufacturer protocol [59]. RNA integrity was verified by electrophoresis and cDNA was synthesized from 0.5 µg of total RNA. Bax, Bcl-2, and Bcl-x_L_ mRNA levels were detected using pre-developed TaqMan primers and probes (Applied Biosystems, Foster City, CA) and run on the ABI StepOnePlus Real-Time PCR system (Applied Biosystems). Samples were run in duplicate and normalized to expression of the endogenous control, glyceraldehyde-3-phosphate. Relative quantification of PCR products were based upon value differences between the target gene and glyceraldehyde-3-phosphate using the comparative CT method.

### Bacterial cultures

Bronchoalveolar lavage (BAL) fluid was obtained by cannulating the trachea and lavaging the lungs with 1 ml of sterile saline. BAL samples were serially diluted in sterile saline and plated on sheep’s blood agar plates. Plates were incubated overnight at 37°C in 5% CO_2_ and colony counts were determined 24 hours later. From plates containing fewer than 300 colonies.

### Myeloperoxidase (MPO) activity

BAL fluid was centrifuged at 5,000 rpm for 5 minutes. Following addition of substrate buffer containing O-dianisidine and 0.0005% hydrogen peroxide, MPO activity was measured at 460 nm wavelengths over 6 minutes (Bio-Tek Instruments-Synergy HT, Winooski, VT). MPO activity was calculated as optical density/minute (U) per µl of BAL fluid.

### Systemic and BAL cytokines

Blood was collected at time of sacrifice, centrifuged at 5,000 rpm for 5 minutes in serum separator tubes and stored at −80°C until use. Both serum and BAL levels of IL-1β, IL-6, IL-10, IL-13, MCP-1, and TNFα were measured by using a multiplex cytokine assay (Bio-Rad) according to manufacturer’s instructions [29]. All samples were run in duplicate.

### Serum and tissue lipids

Blood was collected on the same animals immediately prior to induction of pneumonia and 24 hours later. Serum trigylcerides (TG) and cholesterol levels were determined using commercially available kits (Wako Chemicals, Richmond, VA). Lipid quantitation in the proximal jejunum was performed as previously described [59]. Cholesterol and triglyceride distribution within high density lipoproteins (HDL) and low density lipoproteins (LDL) were quantified following fractionation using fast protein liquid chromatography using tandem Superose 6 columns [65]. Serum bile acid levels were determined using an enzymatic assay kit (Cat No. BQ-092A-EALD, BioQuatnt, San Diego, CA)according to manufacturer’s instructions.

### Liver and splenic pathology

H&E-stained section of liver and spleen were evaluated by a pathologist blinded to sample identity (ABF).

### Statistical analysis

Continuous data sets were tested for Gaussian distribution using the D’Agostino-Pearson omnibus normality test. Comparisons were performed using the Student’s t-test if data were found to have a Gaussian distribution. Alternatively, if data did not have a Gaussian distribution, comparisons were performed using the Mann Whitney test. Data are presented as mean ± SEM. Survival curves were analyzed using the Log-Rank test. All data were analyzed using the statistical software program Prism 4.0 (GraphPad, San Diego, CA), and a p value of <0.05 was considered to be statistically significant.

## Results

### Mortality is worsened in aged septic mice with impaired lipid transport

**Figure 1:**
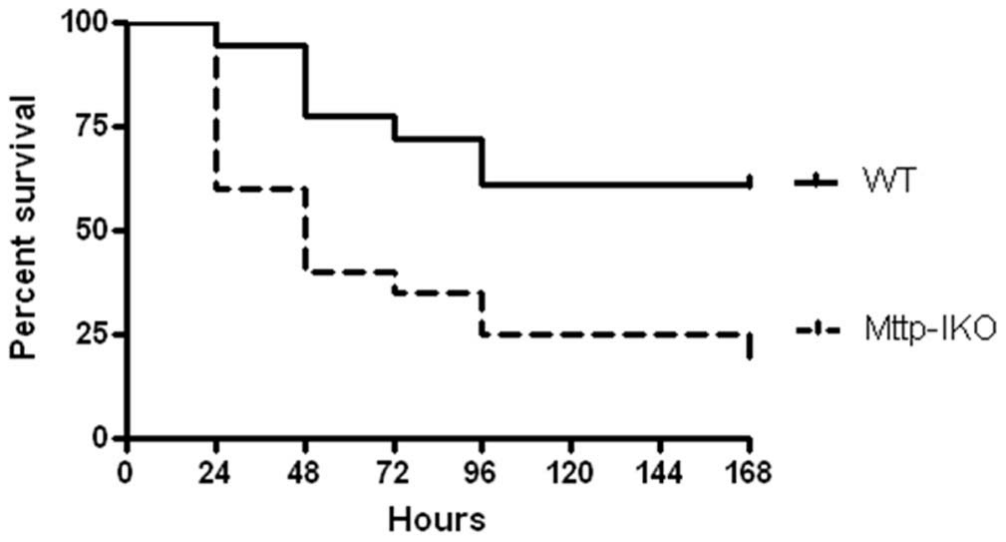
Effect of impaired intestinal lipid transport on mortality following *P. aeruginosa* pneumonia. Mice with intestine-specific deletion of microsomal triglyceride transfer protein had markedly increased mortality compared to WT mice when followed 7 days after induction of sepsis (n = 18–20/group, p = 0.005).

To determine the functional significance of impaired lipid transport, aged WT and *Mttp-IKO* mice were subjected to *P. aeruginosa* pneumonia and followed 7 days for survival (Figure 1). Septic WT mice had a 39% seven-day mortality, while septic *Mttp-IKO* mice had an 80% mortality.

### Intestinal triglyceride mobilization is impaired in aged septic *Mttp-IKO* mice

**Figure 2:**
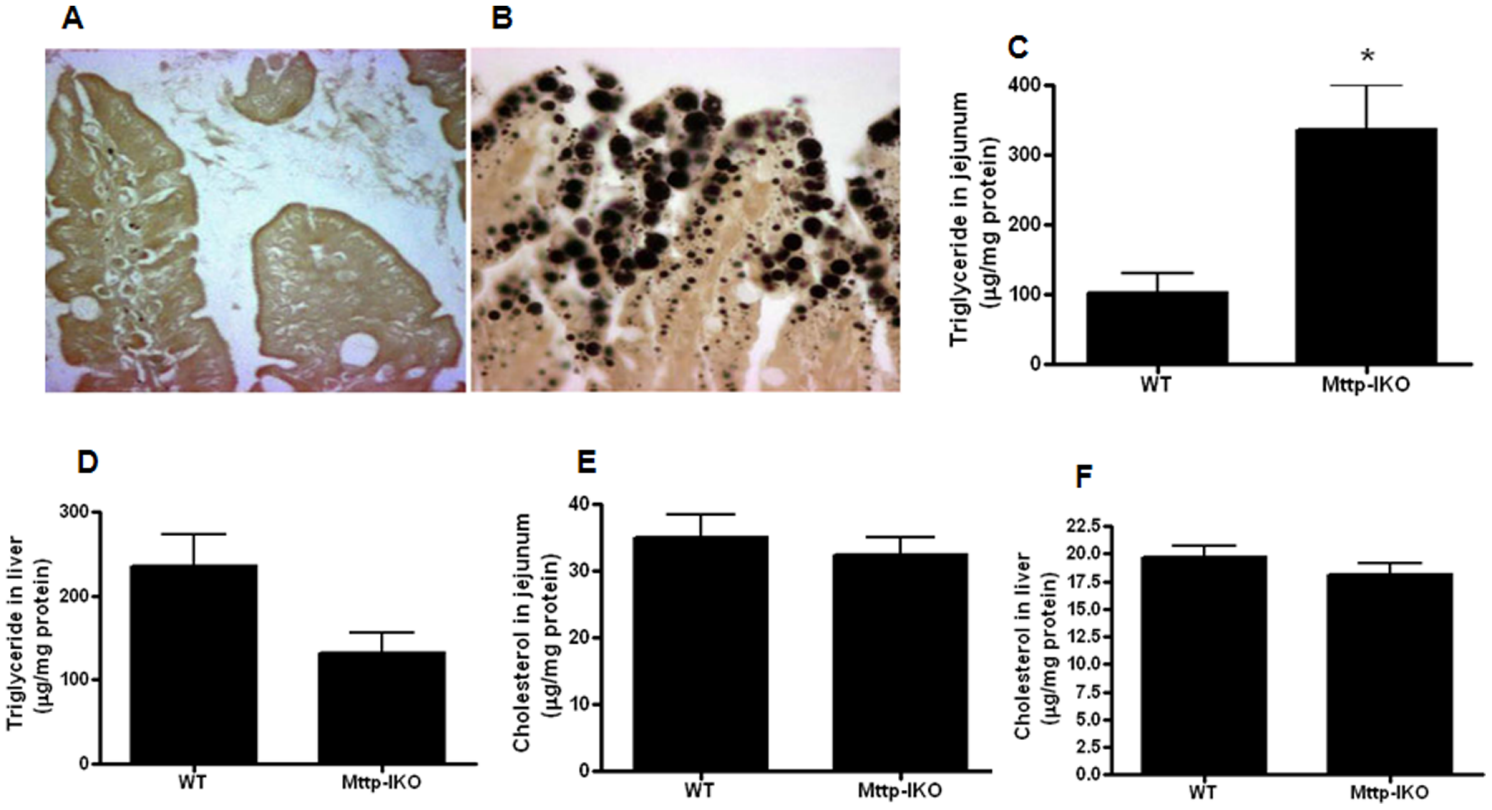
Effect of impaired intestinal lipid transport on intestinal and liver lipids. Villi of aged septic WT (A) and *Mttp-IKO* (B) mice stained with osmium tetroxide to detect intracellular lipid droplets. A massive upregulation is noted in these representative histomicrographs in *Mttp-IKO* mice, (magnification 40x). Triglyceride concentrations were increased in the jejunal mucosa (C, n = 5/group, p = 0.008) but not in the liver (D, n = 5/group, p = 0.056). Cholesterol levels were similar in both jejunal mucosa (E, n = 5/group, p>0.05) and liver (F, n = 5/group, p>0.05).

Defects in chylomicron assembly were associated with large intracellular lipid droplets in aged septic *Mttp-IKO* mice (Fig. 2A, B). Mucosal triglyceride content was increased as expected in aged septic *Mttp-IKO* mice along with the expected decrease in hepatic triglyceride content as previously observed in young Mttp-IKO mice [60], [65] (Fig. 2C, D). By contrast, mucosal and liver cholesterol content was similar between aged septic *Mttp-IKO* and WT mice (Fig. 2E, F).

### Increased Intestinal epithelial apoptosis in aged septic mice with impaired intestinal lipid transport

**Figure 3:**
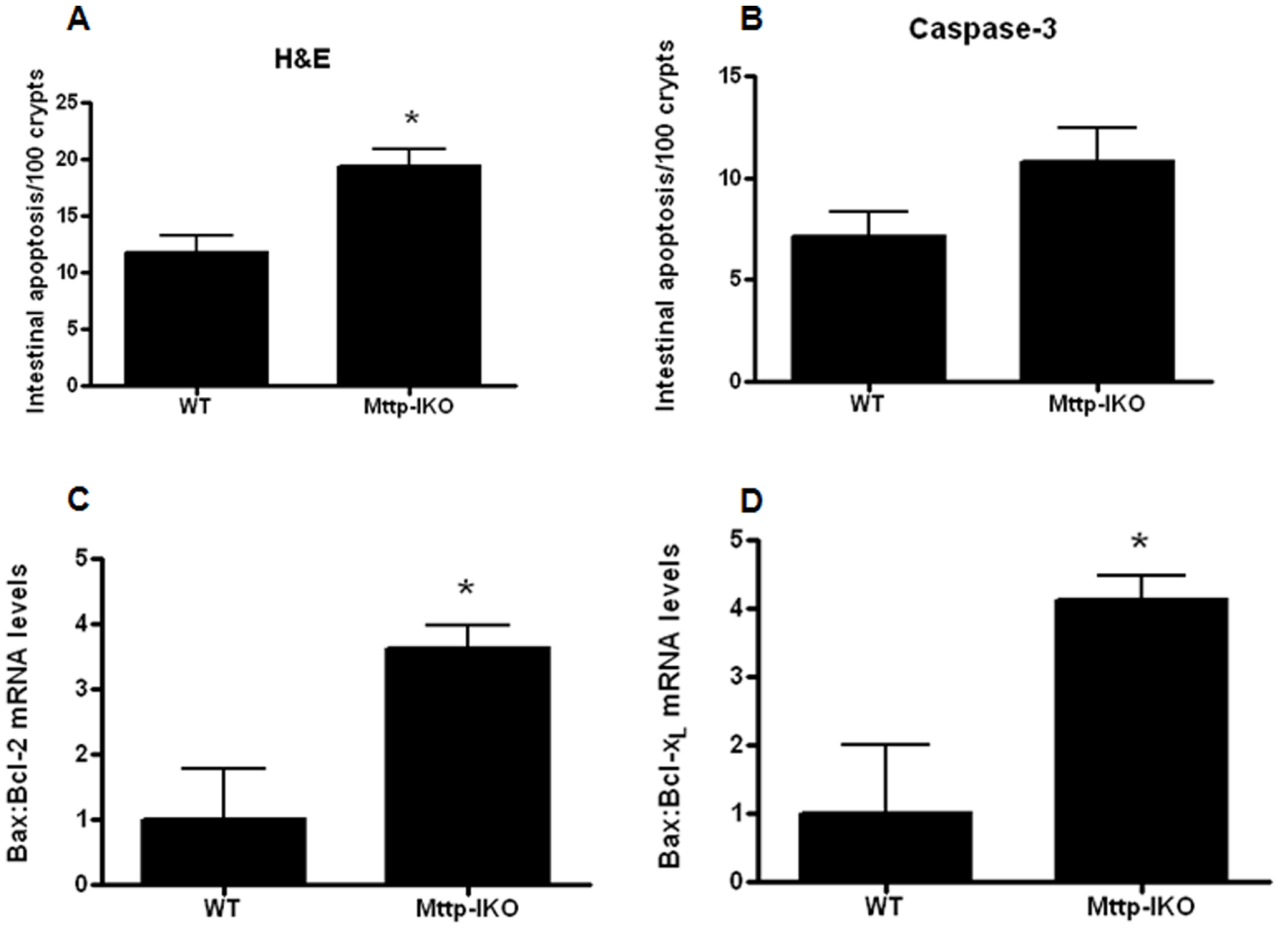
Effect of impaired intestinal lipid transport on intestinal epithelial apoptosis. Intestinal epithelial apoptosis was increased in *Mttp-IKO* mice by H&E staining (A, n = 11–16/group, p = .002) with a similar trend seen with active caspase 3 staining (B, n = 11–12/group, p = 0.07). The ratio of pro-apoptotic Bax to anti-apoptotic Bcl-2 (C, n = 11–16/group, p = 0.02) and Bcl-xL (D, n = 8–11/group, p = 0.004) were both increased in *Mttp-IKO* mice, consistent with elevated apoptosis.

Intestinal epithelial apoptosis was significantly increased in aged septic *Mttp-IKO* mice when assayed by H&E or active caspase 3 staining (Fig. 3A, B). The ratio of jejunal Bax/Bcl-2 (Fig. 3C) and Bax/Bcl-x_L_ (Fig. 3D) was also increased in aged septic *Mttp-IKO* mice compared to aged septic WT mice, consistent with elevated gut apoptosis. These findings are at variance with our prior observations in young septic *Mttp-IKO* mice [59] and suggest that the intestinal apoptotic response to systemic infection is age-dependent.

### Intestinal length and crypt proliferation are higher in aged septic mice with impaired lipid transport

**Figure 4:**
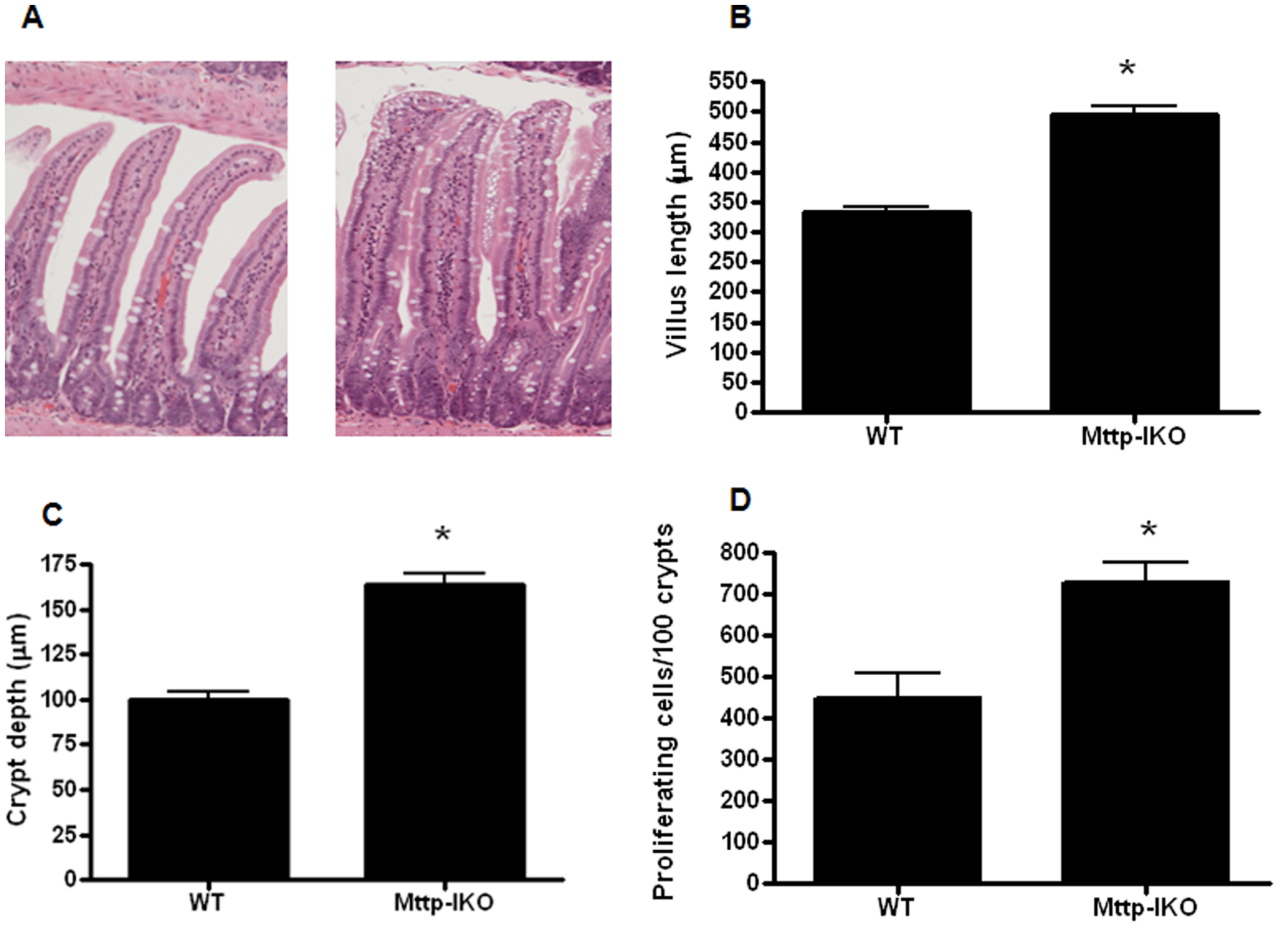
Effect of impaired intestinal lipid transport on villus and crypt length and proliferation. Villus length was longer in in *Mttp-IKO* mice whether assayed qualitatively (A shows representative micrographs, magnification 10X) or quantitatively (B, n = 11–16/group, p<0.0001). Crypt depth was also increased in *Mttp-IKO* mice (C, n = 11–16/group, p<0.0001). The number of s phase cells (D, n = 11–12/group, p = 0.002) was also significantly higher in *Mttp-IKO* mice.

Villus length was significantly increased in aged *Mttp-IKO* mice subjected to pneumonia compared to aged WT mice subjected to the same insult (Fig. 4A, B). Crypt depth was also significantly increased in aged septic Mttp*-IKO* mice (Fig. 4C). This was associated with a significant increase in the number of proliferating S phase crypt cells in aged septic *Mttp-IKO* mice as measured by BrdU staining (Fig. 4D). These findings recapitulate those in young, septic *Mttp-IKO* mice [59], suggesting that elements of the response to systemic infection following attenuated chylomicron formation are retained with age.

### Pulmonary MPO levels are increased in aged septic mice with impaired intestinal lipid transport

**Figure 5:**
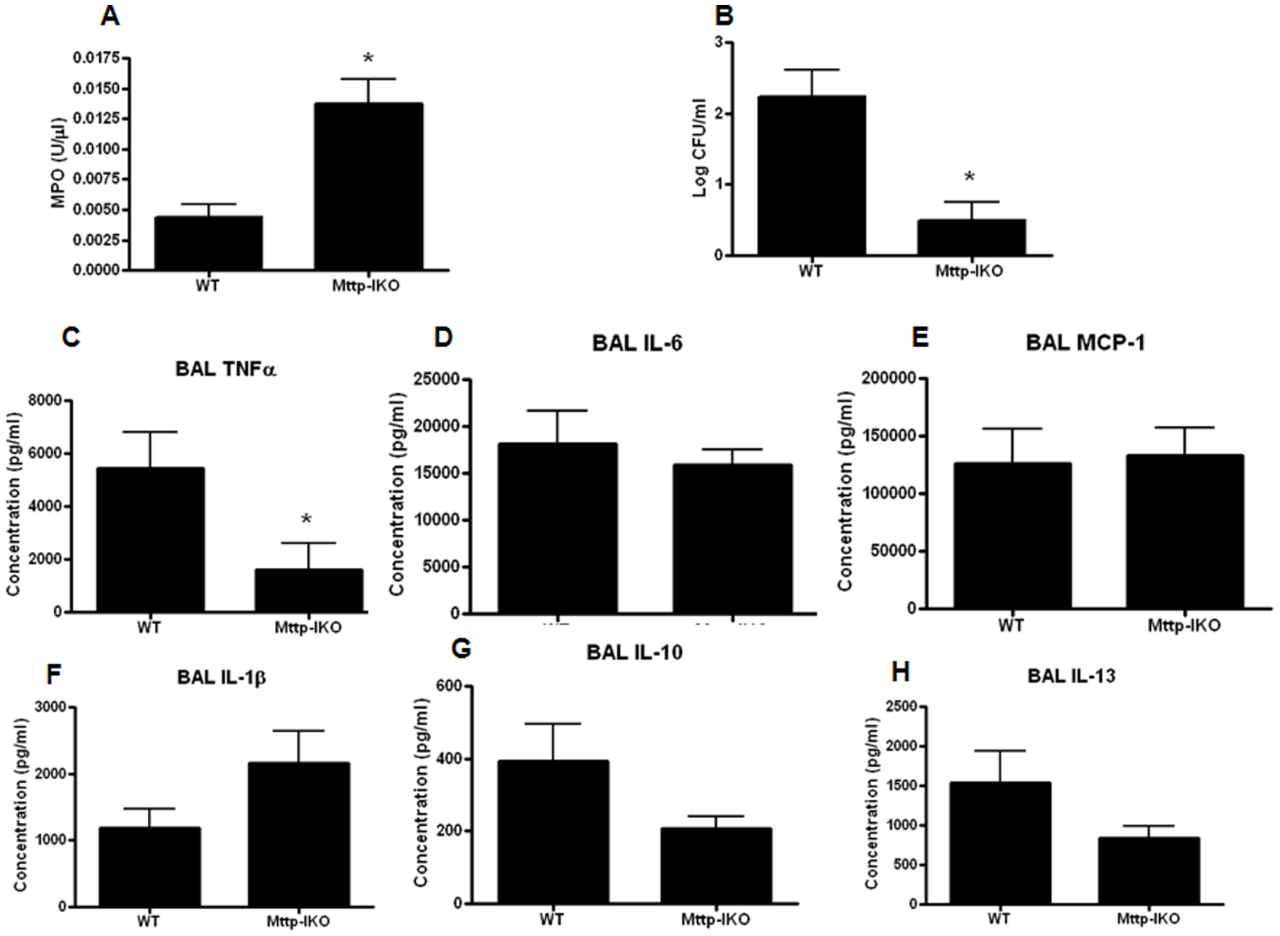
Effect of impaired intestinal lipid transport on BAL MPO, cultures and cytokines. MPO levels were elevated in BAL fluid of *Mttp-IKO* mice (A, n = 8/group, p = 0.001). Bacterial levels were low in both WT and *Mttp-IKO* mice, although the latter had less bacteria present in BAL fluid (B, n = 9–11/group, p = 0.005). TNFα was lower in BAL fluid in *Mttp-IKO* mice (C, n = 7–8/group, p = 0.02) but no statistically significant differences were noted in IL-6, MCP-1, IL-1β, IL-10 and IL-13 (D–H, n = 8–11/group, p>0.05 for each).

Since the infection in pneumonia is initiated in the lungs, pulmonary injury was assayed next. MPO activity was significantly elevated in BAL fluid of aged septic *Mttp-IKO* mice (Fig, 5A). Despite this, bacteria were undetectable in the majority of both septic WT and *Mttp-IKO* mice, and bacterial burden was low even in animals from which *P. aeruginosa* could be recovered (Fig. 5B). Pulmonary cytokines were generally similar between groups except for a decrease in TNFα in aged septic *Mttp-IKO* mice (Fig, 5C–H).

### Systemic pro-inflammatory cytokines are decreased in aged septic mice with impaired intestinal lipid transport

**Figure 6:**
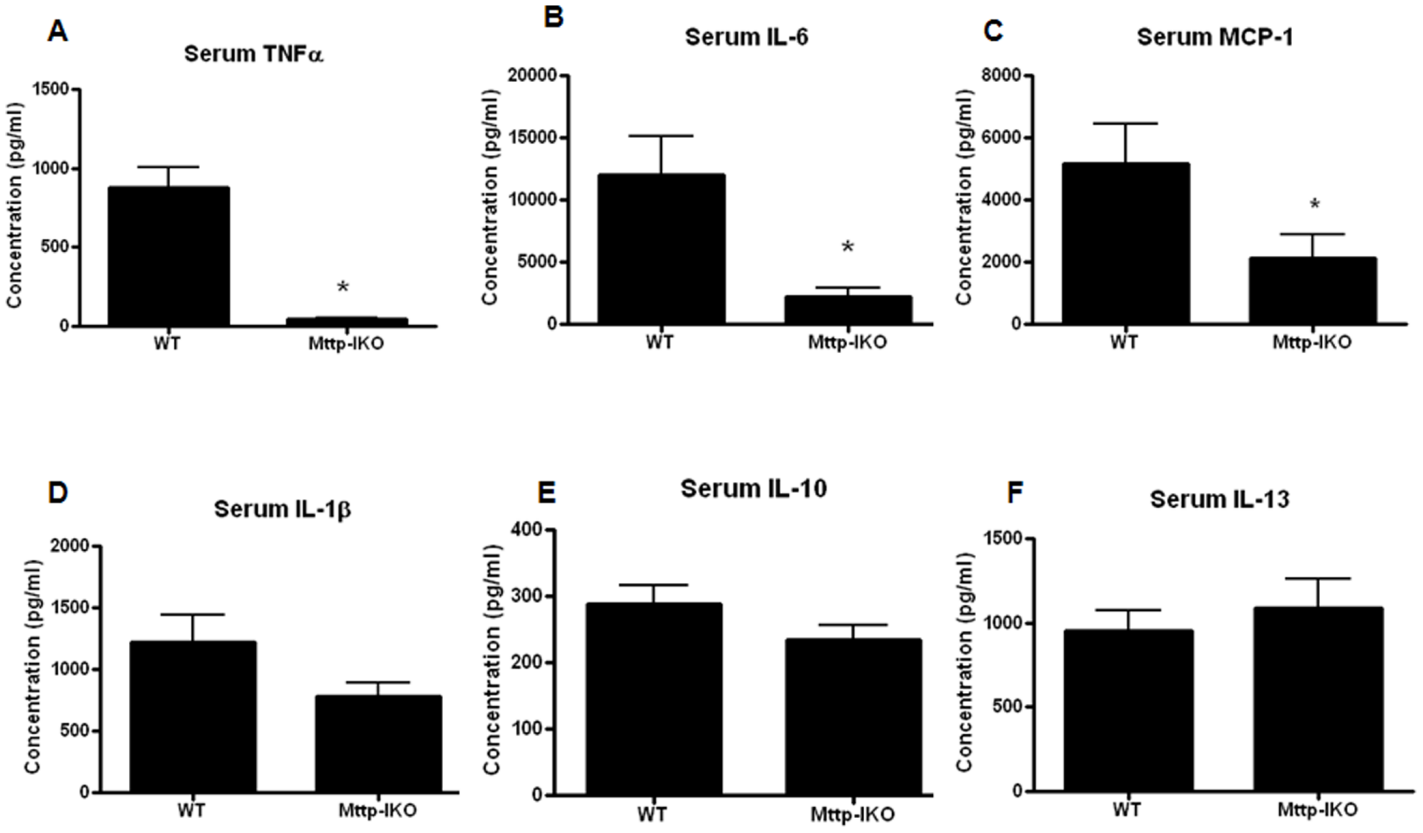
Effect of impaired intestinal lipid transport on systemic cytokines. Serum TNFα (A, n = 9–12/group, p = 0.0001), IL-6 (B, n = 10–14/group, p = 0.008), MCP-1 (C, n = 10–12/group, p = 0.03) were all lower in *Mttp-IKO* mice but no statistically significant differences were noted in IL-1β, IL-10 and IL-13 (D–F, n = 10–16/group, p>0.05 for each).

IL-6, MCP-1 and TNFα levels were all significantly decreased 24 hours after pneumonia in aged septic *Mttp-IKO* mice (Figure 6A–C). In contrast, there were no significant differences in the serum levels of IL-1β, IL-10 and IL-13 by genotype (Figure 6D–F).

### Liver and splenic blastoid cells are increased in aged septic mice with impaired intestinal lipid transport

**Figure 7:**
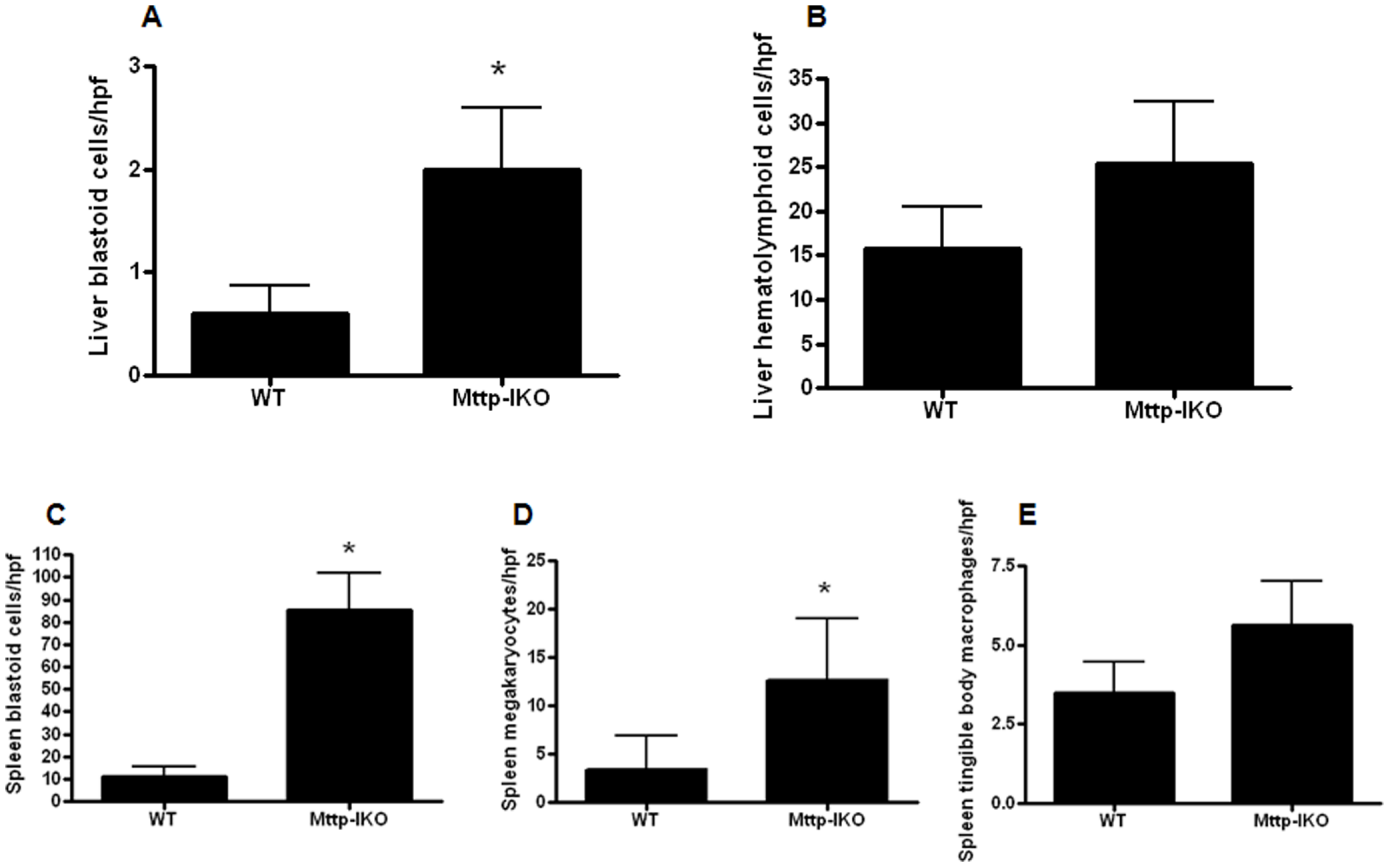
Effect of impaired intestinal lipid transport on liver and spleen cells. In the liver, there was a slight increase in blastoid cells (identified by enlargement, hyperchromasia, and pleomorphism) in *Mttp-IKO* mice (A, n = 10–11/group, p = 0.04) but no difference in hematolymphoid cells (B, n = 10–11/group, p>0.05). In the spleen, there were increases in blastoid cells (C, n = 10–11/group, p = 0.0006) and megakaryocytes (D, n = 10–11/group, p = 0.001) in *Mttp-IKO* mice but no differences in tingible body macrophages (E, n = N = 10–11/group, p>0.05).

The number of liver blastoid cells was increased in aged septic *Mttp-IKO* mice (Fig. 7A), although there were no significant differences in the frequency of liver hematolymphoid cells or enlarged, regenerative hepatocytes (Fig. 7B). Similarly, the number of splenic blastoid cells was also increased in aged septic *Mttp-IKO* mice (Fig. 7D). A marked increase in splenic megakaryocytes was also identified (Fig. 7E) although no difference was detected in splenic tingible body macrophages (Fig. 7F).

### Impact of sepsis, impaired intestinal lipid transport and combination on TG and cholesterol levels

**Figure 8:**
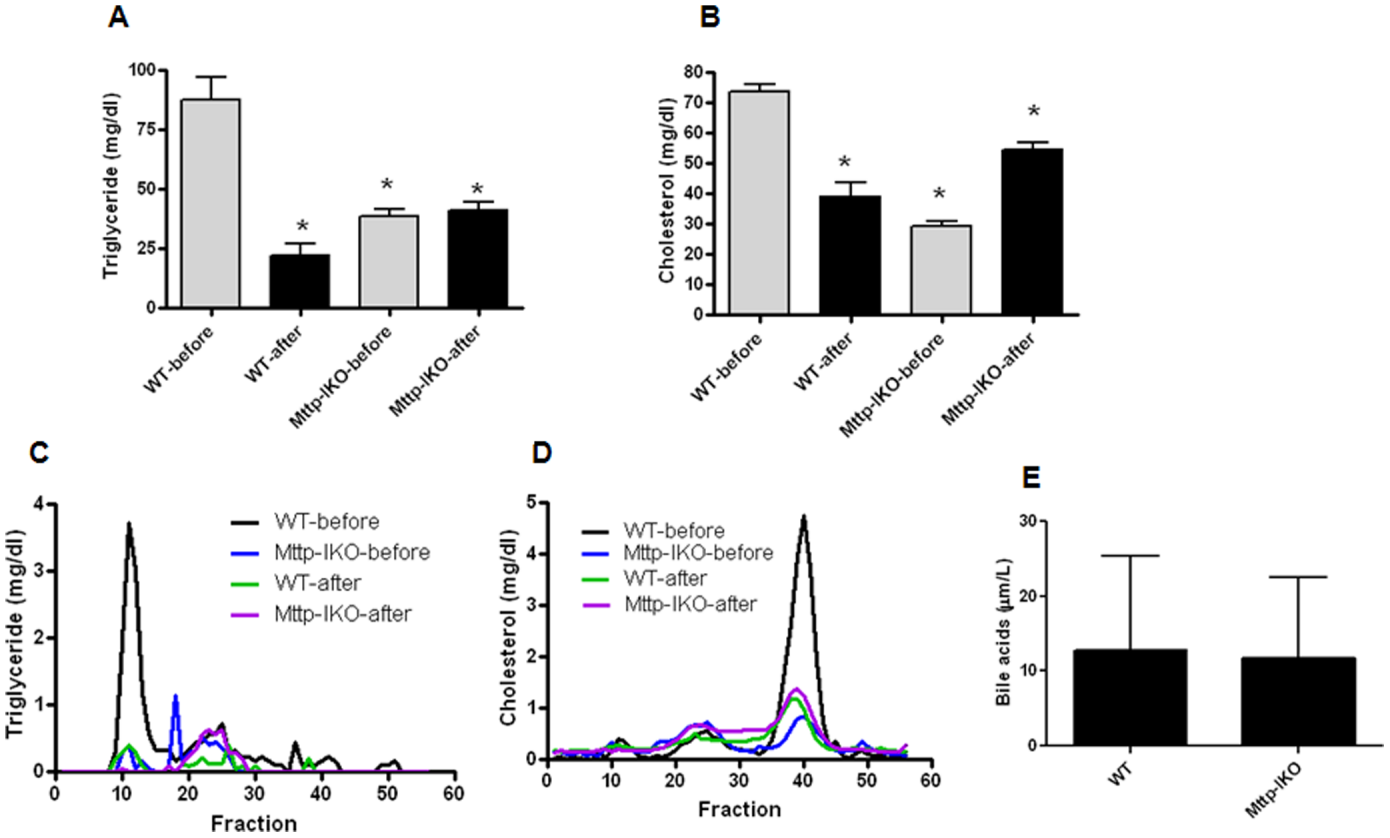
Effect of impaired intestinal lipid transport on serum lipoproteins. Triglyceride (A) and cholesterol (B) in serum were measured before and 24 hours after induction of pneumonia in the same mice. Sepsis decreased both triglyceride and cholesterol after induction of pneumonia in WT mice (C, D, n = 16–18/group, p<0.0001 for both). Inhibiting chylomicron assembly also resulted in lower triglyceride and cholesterol at baseline (n = 17–18/group, p<0.0001 for both). In contrast, sepsis did not alter triglyceride levels in *Mttp-IKO* mice (n = 11–17/group, p>0.05). Additionally, sepsis increased cholesterol levels in *Mttp-IKO* mice after induction of pneumonia (n = 11–17/group, p<0.0001). Sepsis virtually eliminated triglyceride from large, VLDL and LDL size lipoproteins in WT mice (note fractions 10 and 25, respectively). By contrast, Mttp-IKO mice exhibited virtually no triglyceride in lipoprotein particles, as expected. Sepsis resulted in an increase in HDL cholesterol content in *Mttp-IKO* mice (fractions 38–40). Serum bile acids (E) were similar between WT and *Mttp-IKO* mice (n = 7–8/group, p>0.05).

TG and cholesterol concentrations in serum were both decreased by sepsis in WT mice (Fig. 8A, B). Impaired intestinal lipid transport was verified in aged Mttp-IKO mice with reduced serum TG and cholesterol concentrations. However, while TG levels were similar pre- and post- sepsis in *Mttp-IKO* mice, serum cholesterol levels rose modestly after the onset of sepsis in *Mttp-IKO* mice as noted previously in young septic *Mttp-IKO* mice [59]. Lipoprotein distribution as inferred from FPLC fractionation demonstrated that control septic mice have reduced lipid content in large triglyceride rich lipoproteins (VLDL, fraction 10), while septic Mttp-IKO mice showed a rise in HDL cholesterol, as previously noted in young septic *Mttp-IKO* mice [59] (Fig. 8C–D). These findings again verify that important elements of the lipid homeostatic responses noted in *Mttp-IKO* mice are retained in aged mice. Serum bile acids were also examined since previous reports have demonstrated elevated levels in association with liver dysfunction in humans [66]. Bile acid levels were similar between aged septic WT and aged septic *Mttp-IKO* mice (Fig. 8E).

## Discussion

The central finding of this study is that mortality is markedly increased in aged septic *Mttp-IKO* mice following *P. aeruginosa*-induced sepsis. This observation directly contradicts findings in genetically identical young mice where blocking intestinal chylomicron assembly results in a survival benefit. Since the major difference in experimental design between this study and our prior study on young septic *Mttp-IKO* mice [59] is mouse age, it is reasonable to conclude that age plays an important role in the divergent survival in young vs. aged *Mttp-IKO* mice subjected to sepsis. This is consistent with multiple previous studies that have demonstrated that age greatly impacts the host response in sepsis, and interventions that are beneficial in young septic hosts fail to improve or even worsen mortality in aged hosts [4]–[12].

When trying to understand the differential mortality between young and aged septic *Mttp-IKO* mice, it is helpful to compare some of the key physiologic and cellular parameters that we observed. Parameters that are similarly changed between young and aged *Mttp-IKO* mice compared to WT animals – such as villus length, crypt proliferation, pulmonary and systemic cytokines – are unlikely to play a causative role in the differential mortality observed. In contrast, parameters in which results move in the opposite direction (i.e. increased vs. decreased) or change in only one group (i.e. increased vs. no difference) between young and aged *Mttp-IKO* mice animals compared to WT animals could potentially play a role in mediating the differential mortality observed. It is notable, then, that of the multiple parameters examined in this study, only gut apoptosis, pulmonary neutrophil infiltration, systemic TNFΑ, and mucosal triglyceride concentration were different in young and aged septic *Mttp-IKO* mice when compared to WT mice.

Gut epithelial apoptosis is increased in both mouse models and human autopsy studies of sepsis [20], [21], [67]. This appears to be physiologically significant since overexpression of Bcl-2 is associated with increased survival from sepsis from either *P. aeruginosa* or cecal ligation and puncture (CLP) [20], [21]. Numerous co-morbidities including cancer and chronic alcohol abuse are associated with increased sepsis-induced gut epithelial apoptosis. Notably aging is also associated with increased gut apoptosis [29], [33], [34]. Gut apoptosis is increased in aged *Mttp-IKO* mice in this study, associated with increased ratios of both jejunal Bax/Bcl-2 and Bax/Bcl-x_L_. Sepsis in isolation from either methicillin resistant *Staphylococcus aureus* pneumonia or peritonitis increase both pro (Bax) and anti (Bcl-x_L_) apoptotic mediators in the mitochondrial pathway [28], [30], and the ratio of these plays a critical role in determining whether a cell lives or dies. The finding that gut apoptosis is increased in aged septic *Mttp-IKO* mice directly contrasts with young septic *Mttp-IKO* mice, where gut apoptosis was reduced. However, the mechanisms through which blocking intestinal chylomicron alter gut apoptosis are unclear. Further, the association between gut apoptosis and mortality does not distinguish whether gut apoptosis is a mediator of mortality in these studies or simply a marker of illness.

An association also exists between pulmonary neutrophil infiltration and mortality in *Mttp-IKO* mice, with increased MPO activity and mortality in aged septic mice while MPO activity was unchanged in young septic *Mttp-IKO* mice compared to aged matched septic WT mice [59]. Pulmonary neutrophil infiltration is commonly seen in the acute respiratory distress syndrome [68]–[70]. While neutrophils play a beneficial role against pulmonary pathogens, they also release toxic factors such as reactive oxygen species and proteinases that contribute to progression of lung disease. We do acknowledge that is counterintuitive that mice with lower levels of pulmonary bacteria have a higher mortality. However, most mice (both WT and knockout) had no bacteria recovered from the lung 24 hours after the onset of pneumonia and the average bacterial burden was only 100 cfu/ml in WT mice. In the absence of detectable infection in most mice, it is unlikely that the small difference in pulmonary bacterial burden played a major role in mediating higher mortality in aged septic *Mttp-IKO* mice. However, excessive pulmonary inflammation, independent of infection, can lead to self-sustaining damage to lung tissue and then secondarily lead to worsening systemic illness. As such, increased pulmonary neutrophil infiltration represents a plausible potential mechanism underlying mortality in aged septic *Mttp-IKO* mice. Of note, in hemorrhagic shock, toxic mediators are released from the intestine and transported through the mesenteric lymph to the pulmonary circulation. Studies in rats have demonstrated that mesenteric lymph duct ligation prevents lung injury and improves survival in multiple animal models of critical illness [19], [50], [52], [71], [72]. The lungs do not appear to be a major contributor towards improved survival in young septic *Mttp-IKO* mice, mice since decrease of lipid transport into mesenteric lymph did not prevent lung injury. Accordingly, further work will be required to resolve the mechanisms through which blocking intestinal chylomicron alters pulmonary inflammation in an age-dependent manner after sepsis.

One surprising finding was that *Mttp-IKO* mice generally had lower levels of systemic cytokines. Typically, this would be thought of as being associated with better outcomes rather than higher mortality. However, it should be noted that a similar pattern of lower systemic cytokines was seen in both aged and young *Mttp-IKO* mice compared to age-matched WT mice. This suggests that cytokine levels alone cannot be responsible for the differential mortality seen between aged and young mice. It is unclear, however, whether systemic cytokines played no role in survival or if a beneficial role was superseded by a different detrimental mechanism in aged mice. In this regard, it should be mentioned that bacteremia was not different between aged *Mttp-IKO* and WT mice (data not shown).

Patients in the ICU frequently have decreased serum lipids [73]. The functional significance of this observation is not clear. In preclinical models, lipoprotein infusion improves mortality in endotoxemia [74], a model of critical illness with some similarities but also some key differences from sepsis. Alternatively, hyperlipoproteinemic LDL receptor-deficient mice have increased mortality following CLP [75]. It is noteworthy that the adaptive homeostatic responses to sepsis noted in young *Mttp*-IKO mice (increased HDL cholesterol content) appear to be recapitulated in aged mice, but again it is unlikely that this is a determinant of the paradoxical increase noted in mortality.

Another, albeit less likely, factor that could potentially play a role in the differential mortality between young and aged mice is severity of insult. Given that aged mice are more susceptible to sepsis, it is necessary to lessen insult severity in aged animals in order to match mortality between young and aged animals, since aged animals have 100% mortality to sepsis models that cause moderate lethality in younger mice [4]. Both age and severity of insult independently mediate survival and alter the host response following CLP, so it is possible that a differential bacterial burden (rather than age) played a critical role in mediating mortality in this study. Additionally, as alluded to above, while we attempted to match mortality between young and aged mice, baseline mortality from *P. aeruginosa* was 29% in our previous study of young mice whereas baseline mortality from the same organism was 39% in this study. While this difference is relatively small, the risk of death prior to intervention has been shown to play a large role in how (or if) a host responds to a putative sepsis therapeutic [76], [77], and it is possible that this seemingly small difference in baseline mortality was associated with a host milieu that was sufficiently different that blocking intestinal chylomicrons had a profoundly different effect.

This study has a number of limitations. All mechanistic studies were performed at 24 hours, and we therefore do not know how or if any of the parameters altered in aged septic *Mttp-IKO* mice changed over time compared to septic WT mice. This especially limits interpretation of the cytokine data, in light of the fact that cytokine levels are highly dynamic following sepsis [78], [79]. Further, although we compared septic aged matched *Mttp-IKO* and WT mice, it is important to recognize that intestinal *Mttp* deletion was imposed for only 3–4 weeks in these mice. The effects of sustained *Mttp* deletion in aging mice was not examined and it is unknown whether these mice would tolerate virtually lifelong attenuation of lipid absorption. Accordingly, while we previously compared young unmanipulated *Mttp-IKO* and WT mice, it is possible that aging results in local and systemic phenotypic differences between these animals not seen in young mice. As such, some of the differences noted in aged septic *Mttp-IKO* and WT mice might have already been different prior to the onset of pneumonia. It is also important to mention that the mice used were generated from two inbred strains. There is significant controversy as to whether inbred animals are appropriate surrogates for humans which obviously have more genetic diversity [14], [80], [81], and our results must be interpreted in light of the fact that they were derived from two inbred strains. In addition, excision and homogenization of the entire lung would likely have provided a more accurate assessment of pulmonary endpoints examined. Finally, we acknowledge that the associations between increased mortality and the physiologic parameters measured do not imply causation, and additional studies are needed to determine the significance of differences identified between septic aged matched *Mttp-IKO* and WT mice.

Despite these limitations, this study highlights the importance of age in the pathophysiology of sepsis. Further, it demonstrates that blocking intestinal chylomicron secretion can have widely varying results in sepsis, which must be taken into account when considering the role of mesenteric lymph in sepsis. Further research is needed to identify the mechanisms through which blocking intestinal chylomicron assembly alters outcomes in sepsis.
